# Gemcitabine-induced cardiomyopathy: a case report and review of the literature

**DOI:** 10.1186/1752-1947-8-220

**Published:** 2014-06-23

**Authors:** Muhammad F Khan, Silvija Gottesman, Ravichandra Boyella, Elizabeth Juneman

**Affiliations:** 1 Department of Medicine, Southern Arizona VA Health Care System, 3601 South 6th Avenue (SAVAHCS, 1-11C), Tucson, AZ 85723, USA; 2 Department of Medicine, Division of Cardiology, Southern Arizona VA Health Care System, 3601 South 6th Avenue (SAVAHCS, 1-11C), Tucson, AZ 85723, USA

**Keywords:** Gemcitabine, Cardiomyopathy, Cardiotoxicity

## Abstract

**Introduction:**

Newly developed antineoplastic drugs have resulted in improvements in morbidity and mortality from many forms of cancers. However, some of these new chemotherapeutic agents have potentially lethal side effects, which are now being exposed with their widespread use. Gemcitabine is a nucleoside analog, which is a commonly used agent for various solid organ malignancies. Phase 1 and 2 trials with gemcitabine did not show significant risk for cardiotoxicity; however, with its widespread clinical use over the last decade, a few cases of cardiotoxicity related to gemcitabine use have been reported. Cardiomyopathy after the use of gemcitabine monotherapy is extremely rare; and only one such case has been reported in detail previously.

**Case presentation:**

We report a case of a 56-year-old African American man with pancreatic cancer who presented with signs and symptoms of congestive heart failure after being treated with gemcitabine for two cycles (six doses). A two-dimensional echocardiography showed left ventricular ejection fraction of 15 to 20 percent with global hypokinesia. With the absence of significant risk factors for coronary artery disease and a strong temporal relationship with the initiation of chemotherapy, it was concluded that our patient’s cardiomyopathy was related to the use of gemcitabine. Gemcitabine was discontinued and our patient responded well to standard heart failure therapy. Two months later, a repeat echocardiogram showed significant improvements in left ventricular systolic function.

**Conclusions:**

Gemcitabine should be considered as a potential cause of cardiomyopathy in patients receiving chemotherapy with this drug. We need further studies to look into potential mechanisms and treatments of gemcitabine-induced cardiac dysfunction.

## Introduction

Anticancer therapy has emerged tremendously over the last few years. Newly available drugs and protocols have resulted in improvements in morbidity and mortality from many forms of cancers. However, these new antineoplastic drugs and protocols are not without potentially lethal side effects. It is therefore very important to recognize serious toxicities associated with some of these medications, so appropriate measures can be taken to avoid and manage such problems.

Gemcitabine is a nucleoside analog and a pyrimidine antimetabolite that inhibits deoxyribonucleic acid (DNA) synthesis by inhibition of DNA polymerase and ribonucleotide reductase [1]. It is a commonly used antineoplastic for various solid organ malignancies including advanced pancreatic, ovarian, breast, bladder and non-small cell lung cancers [2-7]. The Gastrointestinal Tumor Study Group Phase III trial has shown promising results and has led to the adoption of adjuvant chemoradiotherapy with gemcitabine as the standard of care for advanced pancreatic cancer [6].

Gemcitabine is generally well tolerated and is considered relatively safe as compared to many other chemotherapeutic agents. Its most common toxicities include myelosuppression, changes in gastrointestinal function (nausea, vomiting and diarrhea) and abnormalities in liver and renal function tests [8].

Phase 1 and 2 studies with gemcitabine did not show significant risk for cardiotoxicity, however, with its widespread clinical use over the last decade, a few cases of acute myocardial infarction (AMI) and arrhythmias associated with gemcitabine use have been reported. Here, we report a case of a 56-year-old man with pancreatic cancer who developed dilated cardiomyopathy after being treated with gemcitabine.

## Case presentation

A 56-year-old African American man who initially presented with diarrhea, weight loss and painless jaundice, was subsequently found to have adenocarcinoma of the head of pancreas. An initial workup revealed localized cancer with no evidence of distant metastases. He then underwent pancreaticoduodenectomy with complete surgical resection of a 5cm moderately differentiated pancreatic adenocarcinoma. Although our patient underwent complete surgical resection, a pathological examination revealed a neoplastic invasion of the resected adjacent organs, and one out of the seven resected lymph nodes contained cancer (T3N1M0). At that point, the oncology department recommended to proceed with adjuvant chemotherapy with gemcitabine (1000mg/m2 IV on days 1, 8, 15 on a 28-day cycle for six cycles) to try to reduce the likelihood of recurrence.

However, after completing two cycles (a total of six doses) of chemotherapy, he presented to the emergency department with worsening exertional dyspnea, three-pillow orthopnea, paroxysmal nocturnal dyspnea and fatigue. His physical examination revealed an elevated jugular venous pressure (JVP) (10cm above the sternal angle), bibasilar rales and +2 pitting edema of both lower extremities. Cardiac auscultation revealed a gallop rhythm with an S3 and a grade 3 holosystolic murmur over precordium. A chest X-ray showed cardiomegaly with mild to moderate-sized right-sided pleural effusion. It was thought that his presentation was consistent with fluid overload secondary to congestive heart failure (CHF) and he was started on intravenous (IV) furosemide with partial improvement in his symptoms.

The next day, a two-dimensional echocardiography (2D Echo) was performed, which showed left ventricular ejection fraction (LVEF) of 15 to 20 percent with global hypokinesia along with moderate mitral regurgitation. Given the findings of 2D Echo and the absence of significant risk factors for coronary artery disease (CAD) and ischemic cardiomyopathy (CMP), it was concluded that our patient’s CMP was related to the recent use of gemcitabine. Our patient was then started on carvedilol and an angiotensin-converting enzyme inhibitor in addition to diuretics and he was discharged from the hospital two days later in a euvolemic state.

At that point, the cardiology department recommended stopping further chemotherapy with gemcitabine. The oncology department advised further testing to rule out ischemia as a cause of CMP as, in their opinion, chemotherapy with gemcitabine was the only option to reduce the risk of recurrence in this patient. Two weeks later, our patient underwent myocardial perfusion imaging (MPI), which showed a fixed small- to moderate-sized inferior wall defect without any evidence of active ischemia. The ejection fraction (EF) on MPI was calculated to be around 17 to 20 percent with severe global hypokinesia. Our patient was continued on standard heart failure therapy with one more admission to the hospital for CHF exacerbation about two months later. He responded well to IV furosemide and adjustment of heart failure therapy. A 2D Echo was repeated a few months later and it showed improvement in systolic function with an LVEF of 40 percent.

Due to his poor functional status and underlying CMP, further gemcitabine chemotherapy was stopped. Later, our patient developed a recurrence of his pancreatic cancer; he refused further chemotherapy and decided to proceed with palliative care.

Although the exact etiology of our patient’s dilated cardiomyopathy remains unclear, gemcitabine remains the most likely culprit. The temporal relationship of his symptoms to the initiation of gemcitabine chemotherapy; the lack of risk factors for ischemic CMP and prior history of CAD, finding of global hypokinesia on 2D Echo, absence of ischemia on MPI; and improvement in his systolic function after discontinuation of gemcitabine were all consistent with gemcitabine-induced cardiomyopathy.

## Discussion

Gemcitabine is generally not considered as one of the cardiotoxic agents, and its cardiotoxicity has been very rarely reported in the literature. An overview of phase 1 trials of gemcitabine therapy revealed cardiac arrhythmias in 0.7 to 1.4 percent of the patients, significant reduction in LVEF in 0.2 percent of the patients and the development of exudative pericarditis in 0.2 percent of the patients [9].

Similarly, phase 2 trials of gemcitabine monotherapy did not show evidence of significant cardiotoxicity. To evaluate overall safety of gemcitabine, Aapro *et al.* reviewed 22 phase 2 clinical trials (a total of 979 patients) and found very low incidence of AMI (0.5 percent), CMP (0.4 percent), arrhythmias (0.2 percent) and only one case of mild pericarditis [8]. Among the cardiomyopathy cases, one patient had World Health Organization (WHO) grade 4 toxicity indicating symptomatic cardiac dysfunction not responding to conventional therapy. The other three cases of CHF had WHO grade 3 toxicity, manifesting as symptomatic systolic dysfunction, which responded to therapy [8]. Since the publication of the above mentioned review, we could find two additional cases of heart failure [10,11], four cases of AMI [10,12-14] and one case of supraventricular tachycardia (SVT) [15] in phase 2 gemcitabine trials [Table 1]. In a recent review of the French PharmacoVigilance Database (FPVD), Montastruc *et al.* also found a potential association between dilated cardiomyopathy and chemotherapy with gemcitabine [16].

**Table 1:** Case reports of cardiotoxicity with gemcitabine monotherapy

**Author**	**Demography**	**Dose of gemcitabine**	**Duration of treatment**	**Side effect**	**History of CAD**	**Timing**	**Outcome**	**Probability**
**Ozturk *et al. ***[17]	59F with metastatic leiomyosarcoma	900mg/m2	8 days	Coronary vasospasm	Yes	30 minutes after infusion	Survived. Gemcitabine therapy stopped	Strong
**Bdair *et al*. **[18]	43F with metastatic NSCLC	1000mg/m2	7 weeks	NSTEMI VT	Yes	3 days after infusion	Survived. Gemcitabine therapy stopped	Weak
**Kalapura *et al*. **[19]	54M with metastatic pancreatic cancer	1900mg	5th cycle	NSTEMI	No CAD, no risk factors	6 hours after infusion	Survived. Recurrent symptoms with 7th cycle	Strong
**Tayer-Shifman *et al. ***[20]	67F with metastatic ductal carcinoma of breast	1000-1400mg/m2	3rd cycle	SVT/AVNRT	No CAD, no risk factors	Hours after infusion	Survived. Gemcitabine therapy stopped	Strong
**Santini *et al. ***[21]	78M with pancreatic adenocarcinoma	NK	1st cycle	AF	History of AF in the past	18-22 hours after the infusion	Survived 6 total episodes of AF 18-22 hours after the infusion	Strong
**Ferrari *et al. ***[22]	72F with metastatic lung adenocarcinoma	1200mg/m2	1st cycle	AF	No history of AF	18 hours after the infusion	Survived 3 total episodes of AF each after 18 hours of infusion	Strong
**Ferrari *et al. ***[22]	73F with NSCLC	1200mg/m2	3rd cycle	AF	No history of AF	12 hours after the infusion	Survived. Gemcitabine stopped	Strong
**Tavil *et al. ***[23]	65M with NSCLC	1200mg/m2	2nd cycle	AF	No history of AF	7 hours after the infusion	Gemcitabine stopped	Strong
**Ciotti *et al. ***[24]	70M with metastatic pancreatic adenocarcinoma	NK	1st cycle	AF	No history of AF	6 days after the infusion	AF recurred with further therapy	Strong
**Yajima *et al. ***[25]	82F with advanced pancreatic carcinoma	16, 800mg-total dose	Two years of treatment	HF	NK	2 years	Gemcitabine stopped	Weak
**Khan *et al. *[present case]**	56M with pancreatic adenocarcinoma	1000mg/m2	Two cycles (2 months)	CMP/HF	No prior history of CHF/CAD	2 months	Gemcitabine stopped, partial recovery	Strong

CAD, coronary artery disease; F, female; NSCLC, non-small cell lung cancer; NSTEMI, non-ST elevation myocardial infarction; VT, ventricular tachycardia; M, male; SVT, supraventricular tachycardia; AVNRT, AV nodal re-entrant tachycardia; NK, not known; AF, atrial fibrillation; HF, heart failure; CMP, cardiomyopathy; CHF, congestive heart failure.

Based on the review of medical literature, cardiotoxicity of gemcitabine can be divided into acute toxicities and chronic toxicities.

Acute toxicities include arrhythmias and myocardial ischemia or infarction. In almost all the cases, AMI developed within few hours to a couple of days after gemcitabine infusion. The longest time interval between gemcitabine infusion and the development of AMI was reported to be six days, however in that case, the association of the AMI to gemcitabine therapy was weak. The exact reasons of gemcitabine-induced acute coronary syndrome are not clear, however, authors have speculated a direct endothelial injury resulting in coronary thrombosis or gemcitabine-induced vascular spasm as possible underlying mechanisms. The evidence for prothrombotic and procoagulant effects of gemcitabine comes from several reports of occurrence of vascular events (like thrombotic microangiopathies, strokes, visceral infractions and vasculitides) in association with gemcitabine infusion [26]. Furthermore, in one other study of gemcitabine monotherapy for cutaneous T-cell lymphoma, authors noticed higher incidence of thrombosis and vascular events in patients treated with gemcitabine [10]. Vasospasm was proposed as a cause of chest pain and new-onset left bundle branch block in a patient who developed these symptoms during gemcitabine infusion. Vasospasm then resolved within 10 minutes of antianginal therapy and serological markers for myocardial damage remained negative [17]. Interestingly, all the patients who developed AMI related to gemcitabine therapy had pre-existing CAD. Two of these patients were already on appropriate antianginal medications before they experienced gemcitabine-related myocardial ischemia [17,18]. In another patient with a previous episode of gemcitabine-induced myocardial ischemia, the next gemcitabine infusion was started concomitantly with IV nitroglycerine and beta blockers [19]. However, despite these measures he continued to have recurrent ischemic symptoms with further gemcitabine treatments.

SVTs including atrial fibrillation (AF) have been rarely reported as a manifestation of gemcitabine cardiotoxicity. In contrast to AMI, where most of the patients had underlying CAD, only one of the patients who developed AF related to gemcitabine infusion had previous history of AF [21]. Almost all the SVT episodes developed six to 18 hours after the administration of gemcitabine infusion. Based on this observation, some authors have argued that direct myocardial toxicity from an active metabolite of 2′,2′-difluorodeoxyuridine (which has a half-life of 18 to 24 hours) is likely responsible for these electrical disturbances [21,22]. With the exception of one patient who remained in AF at the time of discharge [22], sinus rhythm could be restored in all of the other patients. Patients who underwent chemical cardioversion (amiodarone or propafenone) converted to sinus rhythm within two hours of onset of AF. Those who were treated conservatively with rate control strategy converted spontaneously to sinus rhythm on the average two days after the presentation. Three patients suffered recurrent AF episodes with further gemcitabine infusions [21,22,24], and two of them had recurrence of AF despite being on prophylactic antiarrhythmics [21,24].

CMP is among the frequently reported cardiotoxicity for many chemotherapeutic agents like anthracyclines, but it has been rarely encountered with gemcitabine monotherapy. The cardiomyopathy seems to develop after a few months to years of treatments with gemcitabine. The exact mechanisms of myocardial damage, and the total dose of gemcitabine required to induce myocardial toxicity are unknown. Most of the patients in phase 1 and 2 clinical trials who developed gemcitabine-induced CMP had underlying CAD. Although our patient did not have known CAD or CAD risk factors, it is still possible that he had a prior silent event (a fixed inferior wall defect on MPI). However, a fixed inferior wall defect did not explain severely depressed left ventricular (LV) systolic function and global hypokinesia seen on 2D Echo. Given the fact that his heart failure symptoms started after the initiation of gemcitabine, we strongly suspected gemcitabine cardiotoxicity as a cause of his CMP. This argument is further affirmed by the fact that his LV systolic function improved significantly after the discontinuation of gemcitabine and the initiation of appropriate treatment.

Gemcitabine has also been implicated in the development of exudative pericardial effusions. Four cases of symptomatic and hemodynamically significant pericardial effusions requiring drainage were reported by Vogl *et al. *[27]. All of these patients were exposed to unblocked cardiac radiations in the past, and all these patients had pericardial abnormalities seen on echocardiography before the initiation of gemcitabine chemotherapy. The authors concluded that gemcitabine therapy in these patients resulted in radiation recall reaction, that is, gemcitabine recalled previous radiation-related inflammation in the pericardium.

## Conclusions

In our opinion, gemcitabine-induced cardiotoxicity should be considered as a potential cause of arrhythmias, ischemia or cardiomyopathy in patients receiving chemotherapy with this drug. We need further studies to look into the potential mechanisms of gemcitabine-induced cardiac dysfunction. In the case of toxicity, discontinuation of further therapy with gemcitabine appears to be the next appropriate step in the management.

## Consent

Written informed consent for publication could not be obtained from the patient as he has died and the next-of-kin could not be contacted despite all reasonable attempts. However, every effort has been made to protect patient anonymity and there is no reason to think that the patient or family would object to publication.

## Competing interests

The authors declare that they have no competing interests.

## Authors’ contributions

MK contributed to the concept/design of the study, data collection, data analysis/interpretation, drafting the manuscript, critical revision of the manuscript, preparation and approval of the manuscript. SG contributed to the concept/design of the study, data collection, drafting the manuscript, approval and preparation of the manuscript. RB contributed to the data analysis/interpretation, critical revision of the manuscript, approval and preparation of the manuscript. EJ contributed to the data analysis/interpretation, critical revision of the manuscript, approval and preparation of the manuscript.
